# Bibliometric analysis of global research trends between gut microbiota and pancreatic cancer: from 2004 to 2023

**DOI:** 10.3389/fmicb.2023.1281451

**Published:** 2023-11-27

**Authors:** Shuang Wu, Su Wen, Kangli An, Liping Xiong, Hao Zeng, Yueyue Niu, Tiejun Yin

**Affiliations:** ^1^Department of Geriatrics, Tongji Hospital, Tongji Medical College, Huazhong University of Science and Technology, Wuhan, China; ^2^Department of Ophthalmology, Henan Provincial People’s Hospital, Clinical Medical College of Henan University, Zhengzhou, China

**Keywords:** pancreatic cancer, gut microbiota, bibliometric, citespace, VOSviewer

## Abstract

**Background:**

Pancreatic cancer (PC) is one of the most lethal malignancies of the digestive system and is expected to be the second leading cause of cancer-related death in the United States by 2030. A growing body of evidence suggests that the gut microbiota (GM) is intimately involved in the clinical diagnosis, oncogenic mechanism and treatment of PC. However, no bibliometric analysis of PC and GM has been reported.

**Methods:**

The literature on PC and GM was retrieved from the Web of Science Core Collection (WoSCC) database for the period from January 1, 2004 to April 25, 2023. Microsoft Excel 2021, CiteSpace, VOSviewer, Scimago Graphica, Graphpad Prism, Origin, the R package “bibliometrics” and the bibliometric online analysis program were used to visualize the publishing trends and hot spots in this field.

**Results:**

A total of 1,449 articles were included, including 918 articles and 531 reviews. Publishing had grown rapidly since 2017, with the 2023 expected to publish 268 articles. Unsurprisingly, the United States ranked highest in terms of number of literatures, H index and average citations. The University of California System was the most active institution, but Harvard University tended to be cited the most on average. The three most influential researchers were Robert M. Hoffman, Zhao Minglei, and Zhang Yong. *Cancers* had published the most papers, while *Nature* was the most cited journal. Keyword analysis and theme analysis indicated that “tumor microenvironment,” “gemcitabine-resistance,” “ductal adenocarcinoma,” “gut microbiota” and “diagnosis” will be the hotspots and frontiers of research in the future.

**Conclusion:**

In summary, the field is receiving increasing attention. We found that future hotspots of PC/GM research may focus on the mechanism of oncogenesis, flora combination therapy and the exploitation of new predictive biomarkers, which provides effective suggestions and new insights for scholars.

## 1 Introduction

Pancreatic cancer (PC) is the seventh most prevalent cancer and ranks fourth in terms of cancer-related deaths globally (Khalaf et al., 2021). In line with American Cancer Society statistics, there will be 64,650 new cases and 54,550 fatalities in the United States in 2023 (Siegel et al., 2023). Anderson Cancer Center estimated that PC is anticipated to surpass breast cancer as the second greatest cause of cancer-related mortality in the United States by 2030 (Rahib et al., 2014). The etiology of PC is not yet fully understood. Age, obesity, genetic factors, alcohol consumption, smoking, chronic pancreatitis and diabetes are regarded as traditional risk factors for PC (Klein, 2021; Zanini et al., 2021; Bennett et al., 2022; Amri et al., 2023; Conway et al., 2023; Michalak and Małecka-Wojciesko, 2023). As PC is characterized by inconspicuous early symptoms and a lack of specific detection indicators, it is often detected in the late stages. Moreover, it is insensitive to conventional and targeted therapies, so it has inferior therapeutic efficacy, a low survival rate and a very poor prognosis (Chouari et al., 2023). The survival rate for patients diagnosed with advanced PC is 30 to 40% at 1 year, less than 20% at 2 years, and only about 5% at 5 years (Yao and Li, 2023). More and more clinicians and researchers are getting involved in pancreatic cancer research, with the aim of finding ways to diagnose and treat the disease at an early stage.

The gut microbiota (GM) is an enormous and diverse community containing trillions of species of bacteria, fungi, and viruses, the most important of which are enteric bacteria that parasitize the intestine and can help their hosts perform a variety of physiological functions (Li et al., 2016; Liu et al., 2021). According to statistics, the human gut is home to over 1,000 different species of bacteria (Ma et al., 2023). With the advances in targeted 16S rRNA pyrophosphate sequencing and metagenomics sequencing, an increasing number of studies have revealed that GM is closely linked to human diseases such as diabetes, cardiovascular diseases, autism, rheumatoid arthritis, and tumors (Hou et al., 2021; Settanni et al., 2021; Xu et al., 2021; Zaiss et al., 2021; Zhao et al., 2022; Feng et al., 2023; Ma et al., 2023). Notably, recent studies have pointed out a substantial link between PC and GM. Researchers have already identified mechanisms by which the GM affects PC, such as modulating the microbe-immune system-tumor axis, modifying the tumor microenvironment, influencing metabolite production, promoting cancer-related inflammation, and altering the efficacy of chemotherapy (Aykut et al., 2019; Dong et al., 2021; Li et al., 2022; Yu et al., 2022; Zhang and Tang, 2022; Hashimoto et al., 2023; Seo and Wargo, 2023). Additionally, these findings point to the GM as a possible diagnostic marker as well as treatment target for pancreatic cancer (Kartal et al., 2022). Thus, GM contributes significantly to the progress of PC.

Bibliometrics is a sophisticated analytical approach for using mathematical and statistical tools to investigate books and other communication media, and it was initially introduced by Alan Pritchard in 1969 (Jiang et al., 2022; Mirhashemi et al., 2022; Sun et al., 2022). Bibliometric analysis, in contrast to traditional systematic reviews, supplies a model for quantitative assessment of academic literature, which allows for quantifying the distribution along with features of academic data from various viewpoints, and thus visualize the knowledge framework, development process, alongside frontier trends in a specific research field (Kokol, 2021; Ge et al., 2022; Lv et al., 2023). The benefit of this approach is that, unlike other major review methodologies, it enables a thorough assessment of an entire subject with thousands of papers (Donthu et al., 2021). Several researchers have analyzed issues pertaining to the connection between cancer and GM using bibliometrics, such as the connection between colorectal cancer and GM (Wu et al., 2023), cancer immunotherapy and GM (Yang et al., 2022), as well as lung cancer and GM (Chen et al., 2023). In the past two decades, more and more clinical and animal studies between PC and GM have started to emerge (Yu et al., 2022; Chen et al., 2023; Takaori et al., 2023). However, there is no quantitative analysis of the literature on studies between PC and GM. Therefore, in an attempt to give the researcher a more comprehensive understanding of the development and current status of the field, we visualized and analyzed various bibliometric indicators by identifying the researches on PC/GM over the last two decades with the help of bibliometric analysis tools. The essential purposes of the analysis were to (1) pinpoint global trends in the field of PC/GM from 2004 to 2023; (2) reveal the top active countries/regions, organizations, and authors within this field; (3) discover the most favored journals for publishing PC/GM-related papers; (4) illustrate the primary research priorities and evolutionary tendencies on PC/GM; (5) predict future research frontiers as well as supply some new perspectives and inspirations for succeeding studies.

## 2 Methods

### 2.1 Data sources

For the literature search in this study, we used the Web of Science Core Collection (WoSCC) database. This database was selected since it is the friendliest and the easiest tool to use for bibliometric research, in addition to contains numerous noteworthy and high-quality journals as well as thorough citation index records (AlRyalat et al., 2019; Liu et al., 2022).

### 2.2 Search strategy

**Figure 1:**
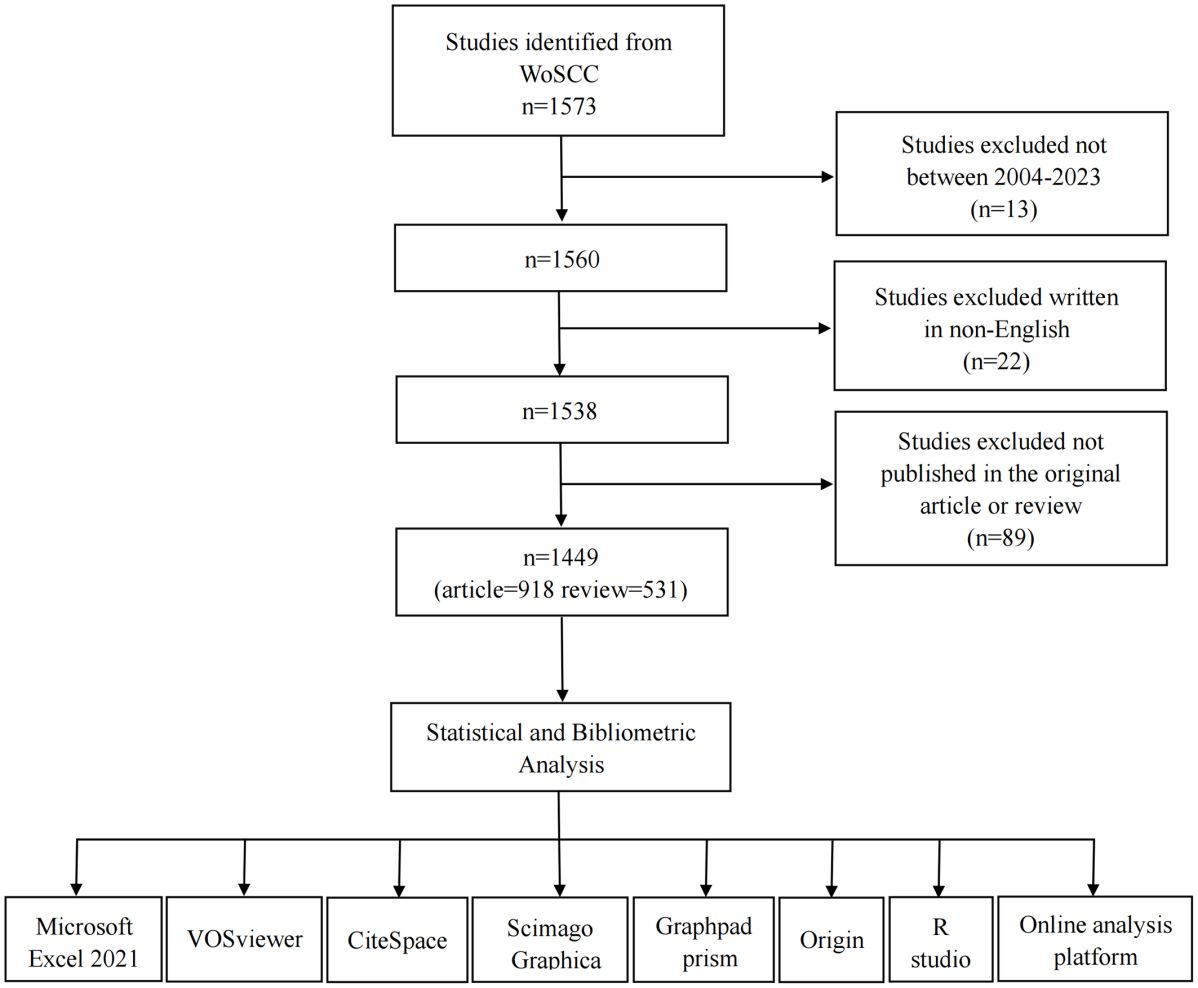
Flowchart of the literature searching and screening in the study.

Listed below was the search strategy: [#1: TS = microflora* OR microbiome* OR flora* OR microbiota* OR bacteria* OR antibiotic* OR dysbiosis* OR probiotic* OR prebiotic* OR Escherichia coli* OR Saccharomyces* OR Bifidobacterium* OR Lactobacillus*; #2: TS = Pancreatic carcinoma OR Pancreas cancer* OR Pancreas neoplasm* OR Cancer of pancreas OR Carcinoma of pancreas OR Pancreatic cancer*; Final dataset: #1 AND#2]. The wildcards (*), which can substitute any other letter and permit keywords with varied ends, enable for the capture of the greatest number of data sources (Cheng et al., 2022a,b). For instance, “neoplasm*” would also return the words of “neoplasm” and “neoplasms.” We made arrangements for two researchers to work independently on the same day to confirm the correctness of the search and publication screening. In cases of conflict, the two researchers conversed and came to a consensus and any remaining disagreements were settled by third negotiations. The inclusion–exclusion criteria for this literature were as follows (Figure 1):

1. The period of literatures was from January 1, 2004 to April 25, 2023;
2. Only articles and reviews literatures were included;
3. The publication language was strictly restricted to English.

### 2.3 Data acquisition

Via the “Export Records as Plain Text” option of WoSCC, all the 1,449 retrieved literature were downloaded with “Full Record and Cited References” for the analysis of bibliometric tools. The yearly number of literatures and citations, authors, nations, organizations, journals, funding agencies, journal impact factor (JIF) and subject category quartile ranks, keywords, references as well as H-index were extracted and hoarded in a Microsoft Excel file. The journal impact factor (JIF) and subject category quartile ranks were acquired fromn. The 2023 Journal Citation Report (JCR, http://clarivate.com/products/web-of-science). H-index, which was defined as the number of papers (h) that had arrived at least h citations, was obtained from the “citation report” function of WoSCC (Szeto et al., 2021). That is to say, an author who has an H-index of 25 has produced 25 articles, each of which received at least 25 citations. The H-index can evaluate the volume and caliber of academic work produced by scholars.

### 2.4 Data analysis

In order to gain more comprehensive data assessment, eight instruments were employed for bibliometric and visualization analysis, including Microsoft Excel 2021, VOSviewer 1.6.19, Scimago Graphica, CiteSpace 6.2.R3, Graphpad Prism 8, Origin 2022, “bibliometrix” package of R 4.3.0 as well as an online analytical platform^1^. Microsoft Excel 2021 was used to produce research flow charts, integrate data from the Web of Science database, in addition to visualising the number of publications and H-indexes for the top 10 funding agencies and the top 5 authors. Scimago Graphica was used to create a more intuitive geographic distribution of national publications using data derived from VOSviewer, where the size of the point represented the number of literatures and the width of the lines connecting the nodes represented the collaboration between the countries (Huang et al., 2023). Graphpad Prism 8 was used to visualise the trend in the number of literatures per year, and to predict the 2023 literatures by fitting a curve to the number. Origin 2022 was used to generate visualisations of the top 20 most frequently occurring keywords. The “bibliometrix” package of R 4.3.0 and an online analytical platform (see footnote 1) was used to generate aesthetically pleasing graphs of the evolutionary trends of the themes. Generally, each point in VOSviewer represented a nation, organization or an author, quantity of literatures defined the size of the point, and the quantity of partnerships determined the magnitude of the linkages between the points (Yeung et al., 2021; Wu et al., 2023). CiteSpace was configured with the following parameters: time span (from January 1, 2004 to April 25, 2023), selection criteria (Top 40), years per slice (Khalaf et al., 2021), link retaining factor (LRF = 3) and g-index (*k* = 15). The g-index is an index based on the number of citations, which can make up for the defect that the h-index cannot reflect the highly cited papers, belonging to the selection criterion in CiteSpace. LRF refers to controlling the number of connections within a certain range (Wang et al., 2023). For example, LRF = 3 means that the maximum number of connections does not exceed three times the number of points.

## 3 Results

In all, 1,573 PC and GM literatures were retrieved from WoSCC. After setting the period from January 1, 2004 to April 25, 2023, eliminating literatures that were neither article or reviews and confining the language used to English, 1,449 literatures were eventually included. The specific article types retrieved are shown in Table 1.

**Table 1:** Types of documents published from 2004 to 2023.

Documents type	NP	Percentage (%)
Article	918	57.74
Review	531	33.4
Meeting abstract	46	2.89
Editorial material	22	1.38
Conference proceedings	21	1.32
Published online	16	1.01
Book chapters	15	0.94
Letter	8	0.5
News item	5	0.31
Recension	5	0.31
Retracted publications	3	0.19

### 3.1 Evolution and growth of literatures

**Figure 2:**
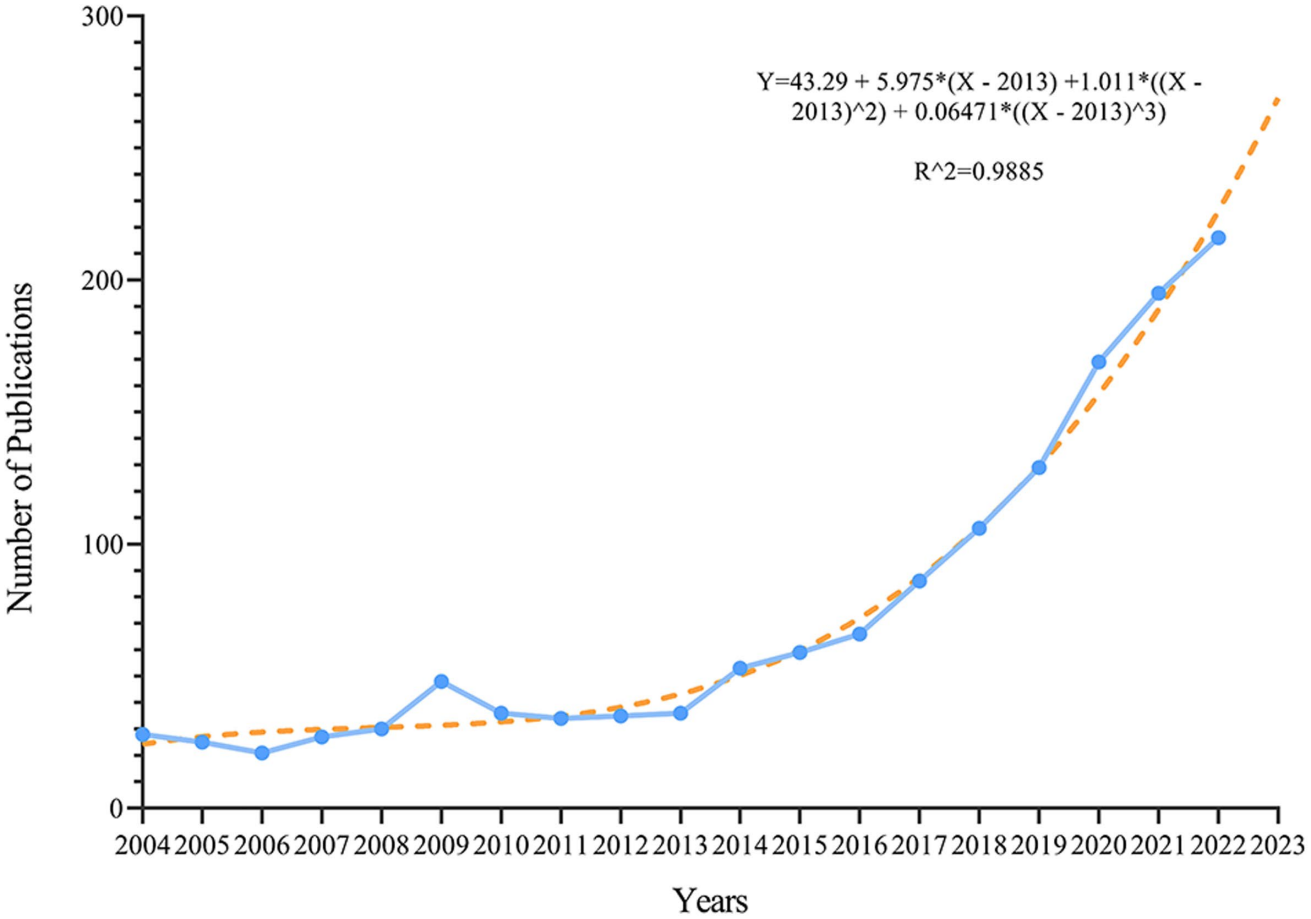
Growth trends of published articles on the PC/GM from 2004 to 2023.

The number of literatures on PC and GM had increased annually over the past 20 years, from 28 in 2004 to 216 in 2022, while the number of literatures in 2023 was lower than in 2022, probably because the search results only included one-third of the year 2023, as shown in Figure 2. The growth of the publication was divided into three phases: the first phase (2004–2013), where the number of literatures grew at a relatively slow and steady rate, and the second phase (2014–2018), where the speed of publishing progress began to increase, while the third stage (2019–2023), the production speed increased sharply. We uncovered a substantial correlation between the number of literatures and the year of publication by matching the data (*R*^2 = 0.9885). Based on the fitted curves, we envisioned that a new maximum of 268 articles on PC and GM will be established by 2023.

### 3.2 Top active countries/regions

**Figure 3:**
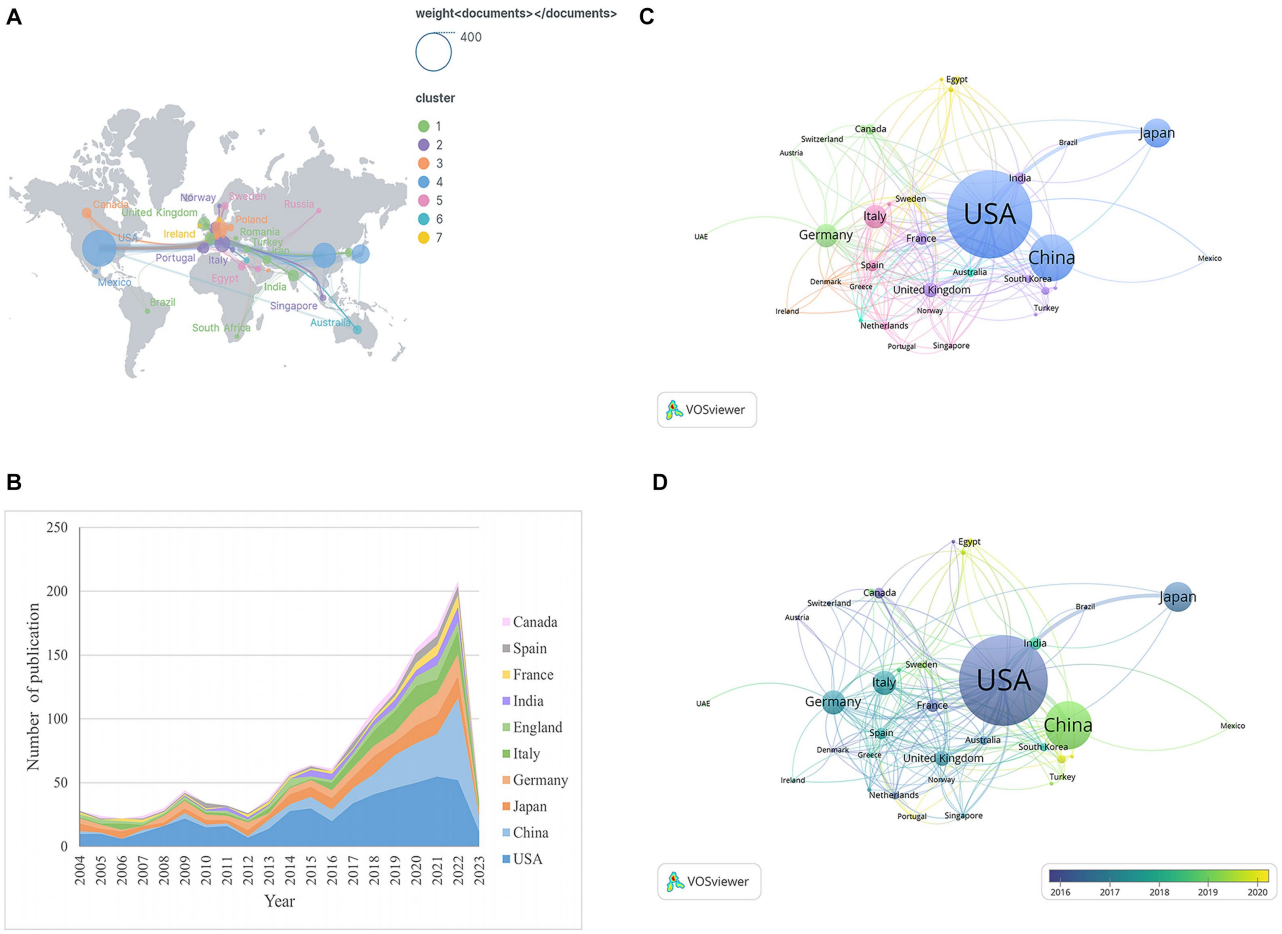
**(A)** The geographic distribution map based on the total literatures of different countries/regions. **(B)** The annual literatures of the top 10 countries/regions from 2004 to 2023. **(C)** The network visualization map of country co-authorship analysis generated by VOSviewer. Each country was represented as a node, and the link between two point indicated co-citation relationship. The larger the node, the more citations were acquired. **(D)** The overlay visualization map of country co-authorship analysis generated by VOSviewer. The node color reflected the corresponding average appearing year (AAY) according to the color gradient in the lower right corner.

In the period 2004 and 2023, 83 countries/regions published to all of the PC and GM literatures. The top ten active nations were inventoried in Table 2, with the USA having the highest number of literatures (*n* = 495, 34.16% of the total), followed by China (*n* = 237, 16.36%), Japan (*n* = 149, 10.28%), Germany (*n* = 122, 8.42%), and Italy (*n* = 115, 7.94%). The USA had the greatest H-index and the most citations on average, far exceeding the other countries by a factor of 77 and 23,084, respectively. Although China published more literatures than Japan, the average number of citations and H-index were lower than in Japan. Figure 3A showed the geographical distribution of PC and GM studies. It can be observed that studies on PC and GM are mainly reported in countries such as North America, Asia and Europe. Figure 3B shows the overall trend of yearly literatures in the top ten nations from 2004 to 2023. Before 2021, the USA had been at the top, but in 2022, China (*n* = 64) surpassed the USA (*n* = 52). As shown in Figure 3C, a visual map of the country collaboration network was constructed using VOSviewer, including countries/regions with a minimum of five documents, and we can visually observe that the USA has the strongest ties to China and Japan. Meanwhile, VOSviewer also performed an overlay visualization map of the country co-authorship analysis (Figure 3D), with the color of the point reflecting each country’s average year of appearance (AAY). In accordance with the color gradient displayed in the bottom right corner, countries marked in green, like China, India as well as South Korea, are relatively new researchers, while the USA, Japan and France were older researchers.

**Table 2:** Top ten active countries.

Rank	Country	NP	Percent	H-index	CPP	TC
1st	USA	495	34.16%	77	46.63	23,084
2nd	China	237	16.36%	38	19.67	4,661
3rd	Japan	149	10.28%	37	32.38	4,824
4th	Germany	122	8.42%	35	28.89	3,525
5th	Italy	115	7.94%	30	29.57	3,401
6th	England	61	4.21%	28	40.56	2,474
7th	India	56	3.86%	18	23.16	1,297
8th	France	51	3.52%	21	35.98	1835
9th	Spain	46	3.17%	18	26.52	1,220
10th	Canada	44	3.03%	19	56.34	2,479

### 3.3 Top active institutions and funding agencies

**Figure 4:**
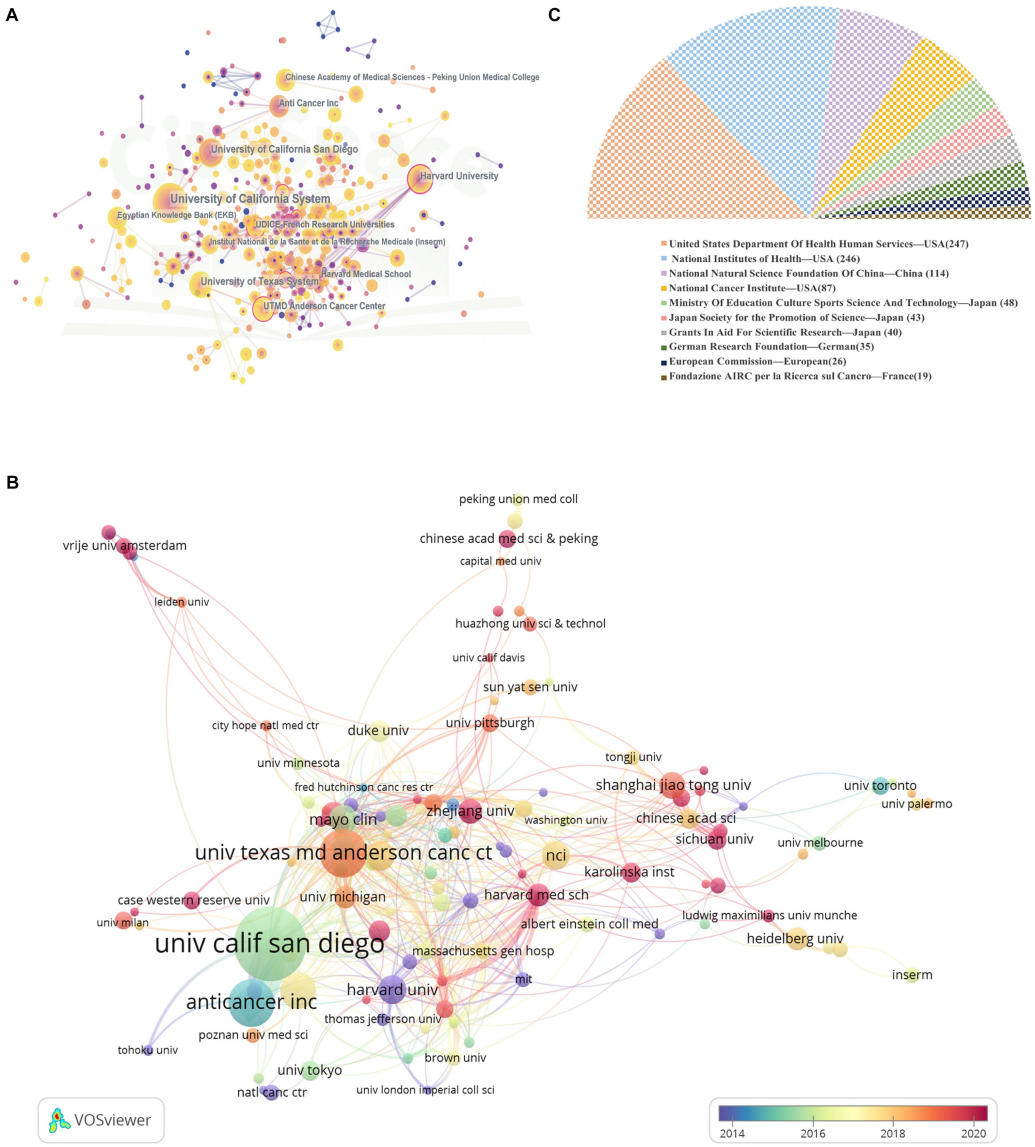
**(A)** The network visualization map of journal co-authorship analysis. **(B)** The overlay visualization map of journal co-authorship analysis. **(C)** The top 10 most active funding agencies involved in this field.

The research of PC and GM covered 2072 organizations in total. The University of California System contributed the majority of articles (*n* = 85) among institutions (Table 3), followed by Research Libraries UK, RLUK (*n* = 51), University of California San Diego (*n* = 44), the University of Texas System (*n* = 43), and Harvard University (*n* = 36). Seven of the top ten active organizations were in the USA, two were in France, and one was in the United Kingdom. It is worth noting that although Harvard University was in fifth place for total production, it had the highest CPP. CiteSpace carried out the analysis of organization collaboration. Each node embodied an organization, the size of the node was equal to the number of papers, while the lines linking these points indicated collaborative links, as illustrated in Figure 4A. These outermost points in this figure with purple circles denote higher centrality (centrality >0.1). Thus, institutions such as UTMD Anderson Cancer Center, Harvard University, AntiCancer Inc., and UDICE-French Research Universities, occupied a central position in the collaborative network. VOSviewer performed an analysis of institutional collaborative authorship (Figure 4B). Only 133 institutions with at least 5 literatures were included in the study. The color gradient in the lower right corner indicates that these institutions such as Sichuan University, Chinese Acad Med Sci&Peking, Havard Med Sch, Univ Tehran Med Sci, Ludwig Maximilians Univ. Muncher, China Med Univ., Zhejiang Univ., case western reserve Univ. are colored red with larger AAY values, on the contrary, Havard Univ., Columbia Univ., Albert Einstein Coll Med, Shanghai Jiaotong Univ., and Thomas Jefferson Univ. are given blue color with AAY with smaller values. In addition, we summarized the top ten active funding agencies in the industry (Figure 4C). The United States Department of Health and Human Services (*n* = 247) had the most literatures, followed by the National Institutes of Health (*n* = 246) and the National Natural Science Foundation of China (*n* = 114). The United States, China, and Japan provided the majority of the financing for this study field.

**Table 3:** Top ten active institutions.

Rank	Institutions	Countries	NP	H-index	TC	CPP
1st	University of California System	USA	85	35	4,830	56.82
2nd	Research Libraries UK, RLUK	UK	51	28	2,396	46.98
3rd	University of California San Diego	USA	44	27	2,314	52.59
4th	University of Texas System	USA	43	18	3,552	82.6
5th	Harvard University	USA	36	21	3,701	102.81
6th	UT MD Anderson Cancer Center	USA	31	16	3,013	97.19
7th	UDICE French Research Universities	France	29	14	1,152	39.72
8th	AntiCancer Inc	USA	27	10	1,109	41.07
9th	Institut national de la santé et de la recherche médicale	France	27	13	713	26.41
10th	Harvard Medical School	USA	24	14	2,126	88.58

### 3.4 Top active authors

**Figure 5:**
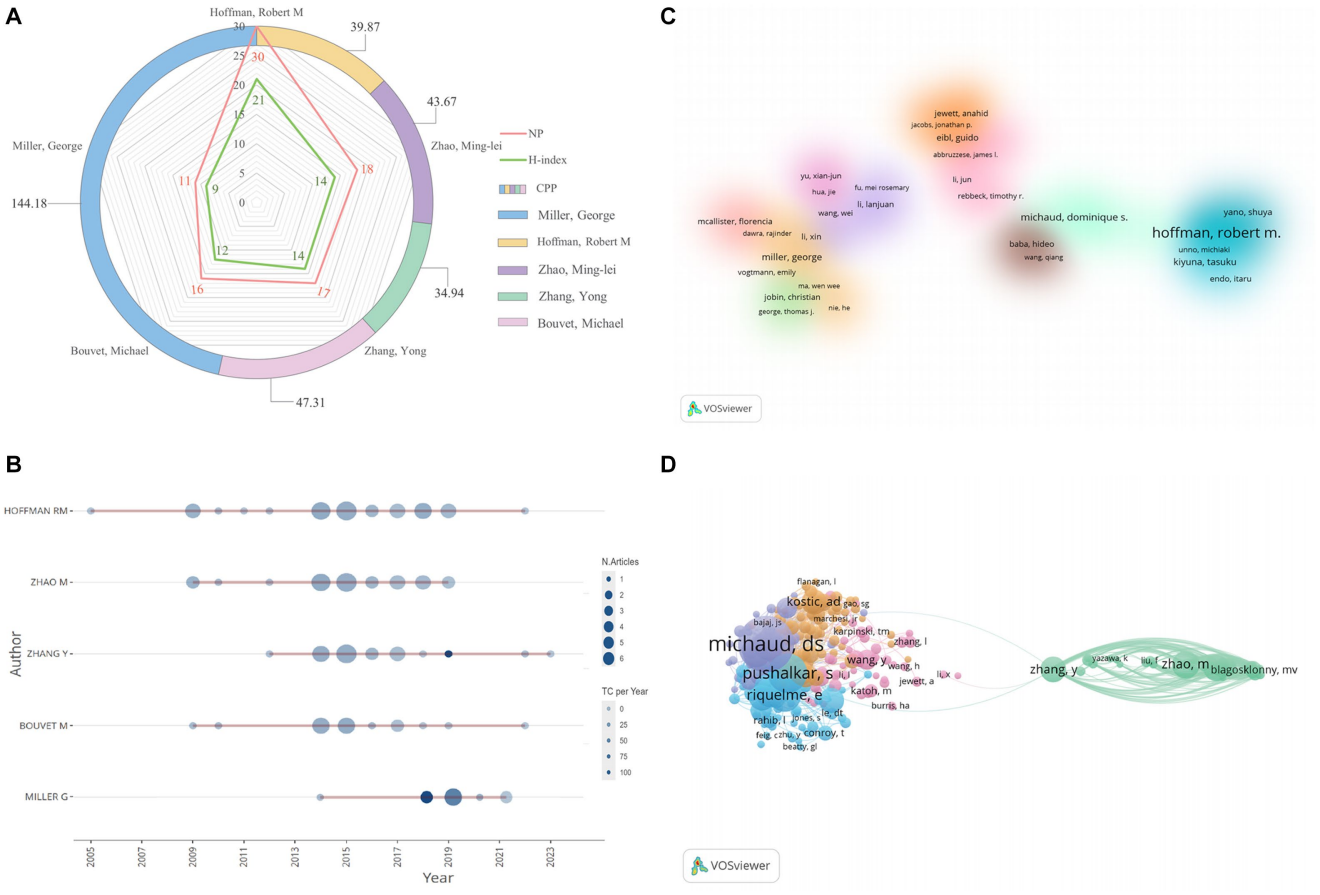
**(A)** The total number of literatures, average citations per document, and H-index of top 5 authors in this domain. **(B)** The top 5 authors’ production over time. The circle size represented the number of documents, and the shade of the color signified the total number of citations. **(C)** The cluster visualization map of author cooperation analysis. Authors with close collaborative relationships are assigned to the same cluster with the same color. **(D)** The network visualization map of author co-authorship analysis.

In regard to author analysis, Figure 5A displayed the top 5 active authors. Robert M. Hoffman (*n* = 30) had the most literatures, followed by Zhao Ming-Lei (*n* = 18) and Zhang Yong (*n* = 17). In addition, Robert M. Hoffman was also the author with the highest H-index. Interestingly, George Miller (*n* = 9) had the greatest average amount of citations, with 144.18, while only placing fifth in numbers of literatures. Figure 5B showed the yearly output and citations of the top five authors for the period 2004 to 2023. The cluster density plot for the author collaboration analysis was also depicted in Figure 5C, including only authors with more than 2 papers. In total, 12 author clusters were formed. 233 authors with at least 25 citations were included in the author co-citation analysis, as can be seen in Figure 5D. With regard to total link strength (TLS), the top 3 authors were Michaud DS (8598), Pushalkar S (5486), and Fan XZ (4468).

### 3.5 Top active journals

**Figure 6:**
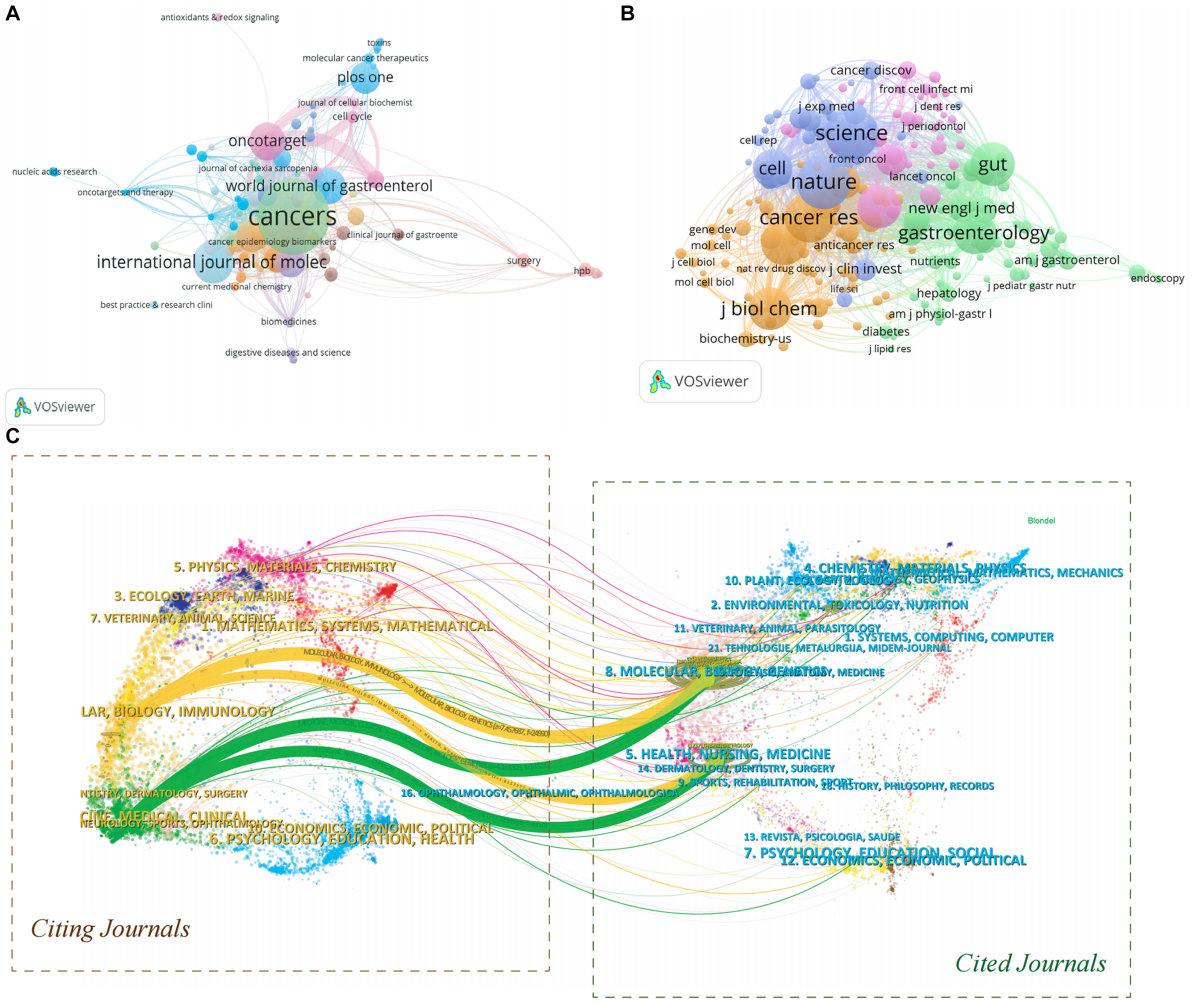
**(A)** The network visualization maps of citing journals. **(B)** The network visualization maps of co-cited journals. **(C)** The dual-map overlay of journals related to PC/GM from 2004 to 2023 by CiteSpace.

As an important vehicle for scholarly activity, journals are crucial in the content indicators of academic evaluation. The journals with the most literatures in a given field can provide suggestions for the dissemination of findings. We found 721 journals assessing research in the field. Table 4 showed details on the top ten journals according to the quantity of papers. Among them, *Cancers* (*n* = 50) had the most articles published in the area, followed by *International Journal of Molecular Science* (*n* = 25) and *Onco Targets and Therapy* (*n* = 22). Among the top ten active journals, *Frontiers in Immunology* had the highest IF (8.876), *Scientific Reports* had the highest total citations (1408), as well as *Cancers* had the highest H-index (Settanni et al., 2021). In Journal Citation Reports (JCR), 90% were categorized as Q2 or Q3. In addition, VOSviewer produced a network visualization map of citing and co-cited journals as depicted in Figures 6A,B. The minimum number of citations was set to 100, and a total of 202 co-cited journals were included. With a TLS of 232,429, *Nature* received the most co-citations, ahead of *Science*, *Cancer Res*, *Proc Natl Acad Sci U S A*, and *Gut*. As shown in Figure 6C, we also created a double map overlay of the journals through CiteSpace to demonstrate their subject dispersion and transfer paths. Two major color lines were used to represent citation linkages between citing and co-cited journals.

**Table 4:** Top ten active Journals.

Rank	Journal	Country	NP	H-index	TC	IF (2022)	JCR
1st	Cancers	Switzerland	50	17	1,198	6.575	Q2
2nd	International Journal of Molecular Science	USA	25	15	560	6.208	Q2
3rd	Onco Targets and Therapy	UK	22	13	738	4.345	Q3
4th	World Journal of Gastroenterology	China	20	12	569	5.374	Q2
5th	Frontiers in immunology	Switzerland	18	11	333	8.786	Q2
6th	Frontiers in oncology	Switzerland	18	9	539	5.738	Q3
7th	PLoS One	USA	18	9	324	3.752	Q3
8th	Scientific Reports	UK	18	9	1,408	4.996	Q3
9th	Nutrients	Switzerland	15	9	379	6.706	Q2
10th	PANCREAS	USA	12	8	1,025	3.243	Q4

### 3.6 References and co-cited references analysis

**Figure 7:**
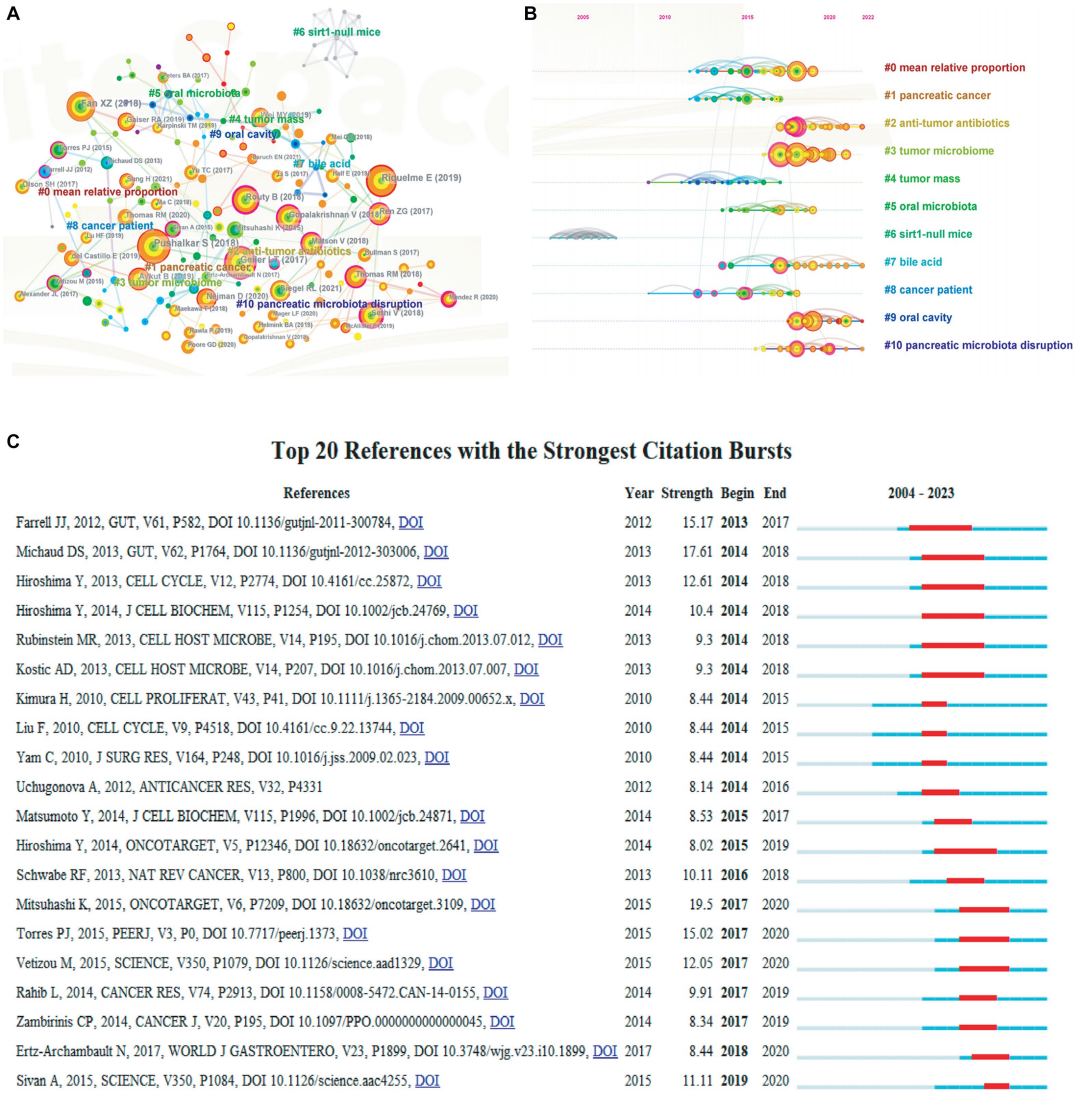
**(A)** The cluster view map of reference co-citation analysis generated by CiteSpace. **(B)** The timeline view map of reference co-citation analysis generated by CiteSpace. **(C)** The top 20 references with the strongest citation bursts generated by CiteSpace. The bars in blue represented the timeline; the bars in red represented a burst period of the references.

Supplementary Table S1 detailed the features of the top ten most cited papers within studies related to PC and GM. The top ten literatures were all cited exceeding 380 times, and four of them were cited in excess of 600 times. The most cited paper was “Mucins in the mucosal barrier to infection,” published by Linden, S. K in *Mucosal Immunology*. It is noteworthy that 80% of the literature types were articles. A reference co-citation network with 11 clusters was created by means of CiteSpace (Figure 7A). Where the modularity, Q was 0.9091 and mean silhouette, S was 0.976, indicating that the clustering of the network was significant and reliable. Based on this, we generated a timeline representation based on co-citation clustering (Figure 7B), which was a way to visualize data combining clustering and time-slicing techniques, aiming to express the chronological features of research hotspots in that research area. With the points on the left representing more ancient citations and the points on the right representing more contemporary citations, distinct colors of the points on the same line denoted various years. Obviously, #9oral cavity and #10pancreatic microbiota disruption, located at the leftmost end of the line, were early research topics in the field. On the contrary, #0mean relative proportion, #1pancreatic cancer and #2anti-tumor antibiotics were the current new research focus in the field. To better comprehend the evolution of research on PC and GM, we also explored the top 20 references that experienced strong citation outbursts implementing CiteSpace (Figure 7C). The first cited burst occurred in 2013 and was published by Farrell et al. in 2012 (Farrell et al., 2012). Mitsuhashi et al. in 2015, Michaud et al. in 2013 and Farrell et al. in 2012 had the greatest burst strength (19.5, 17.61, and 15.17) (Farrell et al., 2012; Michaud et al., 2013; Mitsuhashi et al., 2015), the most recent burst occurred in 2019.

### 3.7 Keywords and themes analysis

**Figure 8:**
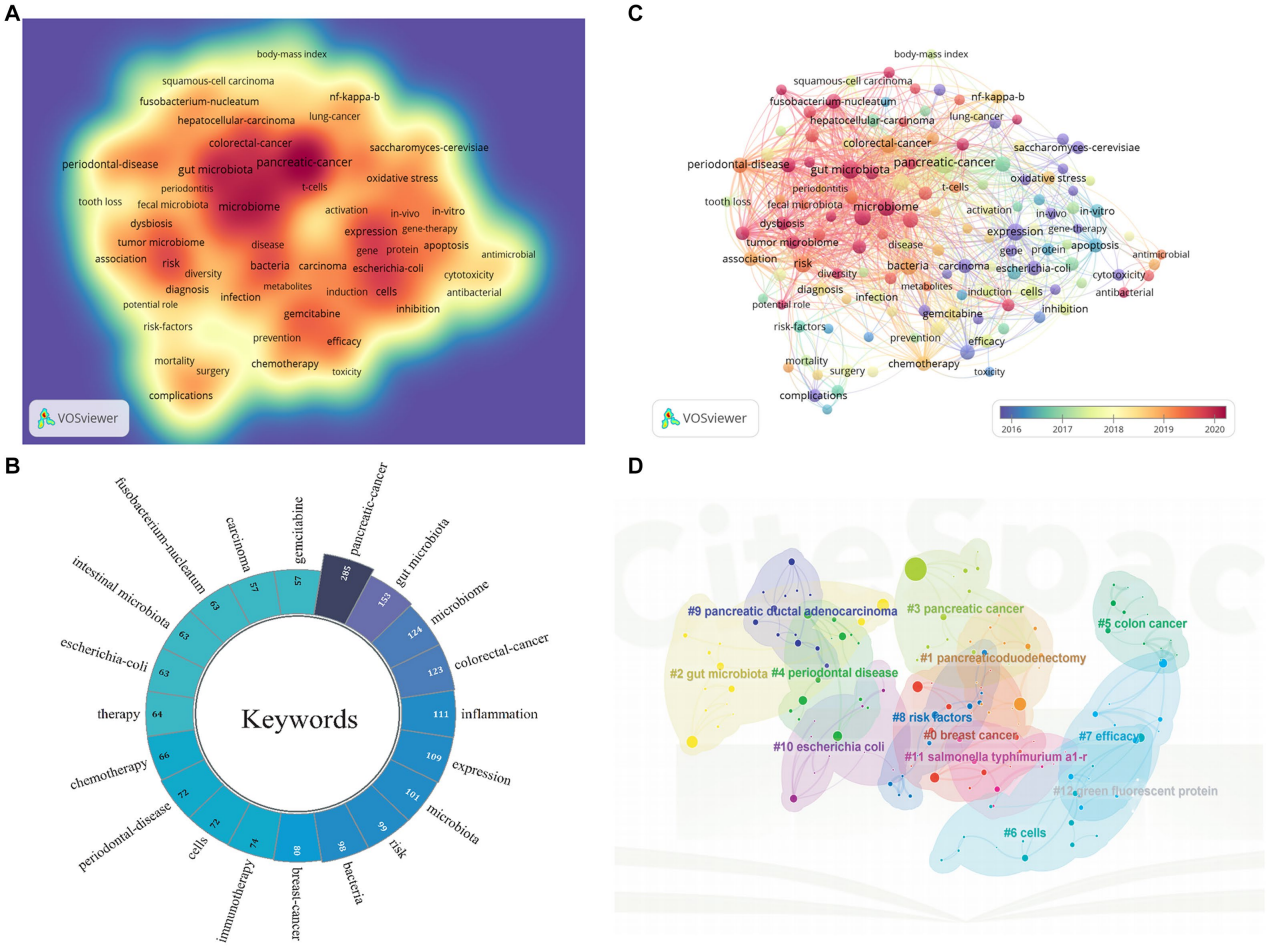
**(A)** The cluster visualization map of keywords analysis. **(B)** The top 20 keywords appearing over time. **(C)** The timeline view map of keywords analysis generated by CiteSpace. **(D)** The cluster view map of keywords analysis generated by CiteSpace.

**Figure 9:**
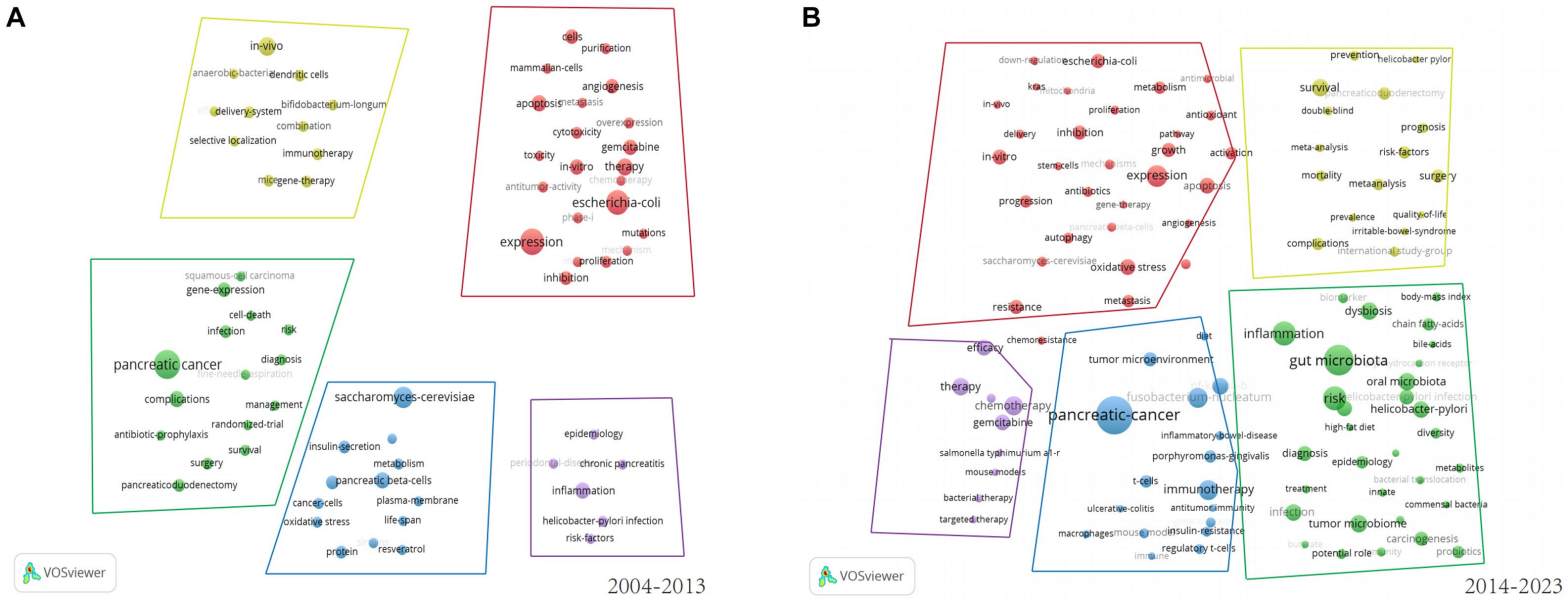
**(A)** The keywords landscape for the period from 2004 to 2013. **(B)** The keywords landscape for the period from 2014 to 2023.

**Figure 10:**
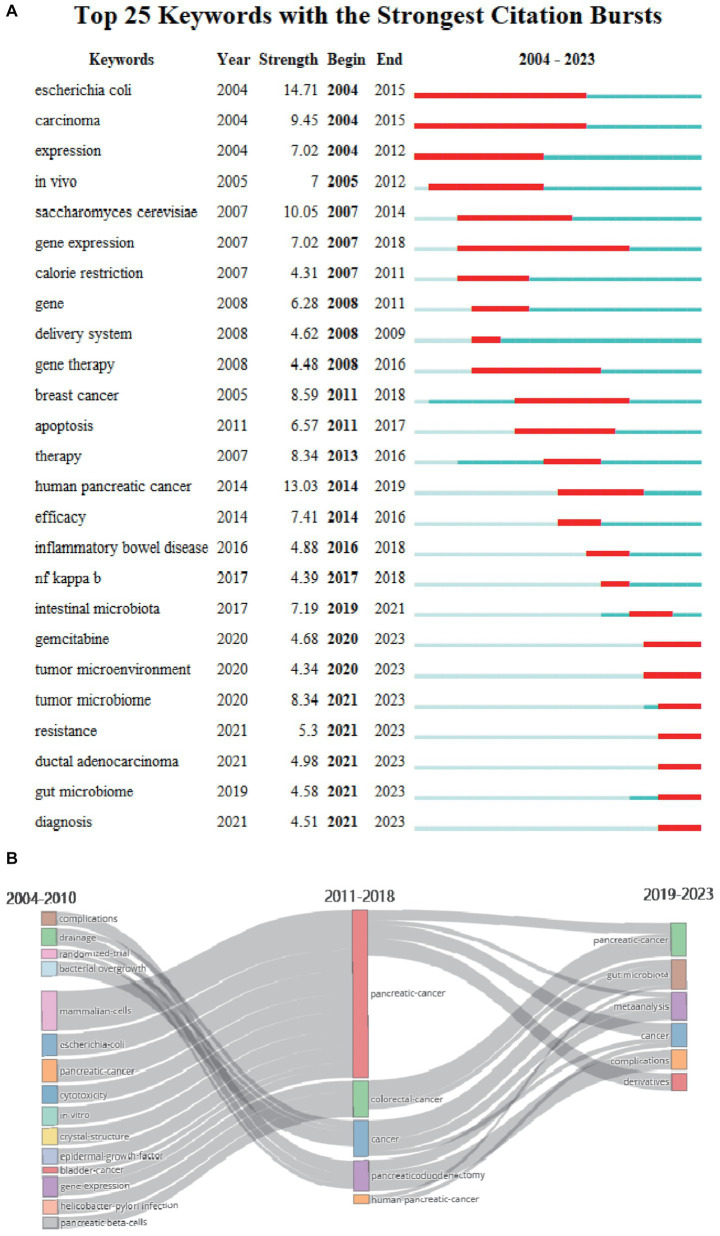
**(A)** The top 25 keywords with the strongest citation bursts generated by CiteSpace. **(B)** Thematic evolution of the three stages in this field.

Keywords are the core of a paper and trend assessment of high-frequency keywords might reveal cutting-edge knowledge in particular fields, so keywords analysis is necessary. The study created a visual map of keyword density utilizing VOSviewer (Figure 8A), and after eliminating nonsensical keywords and combining synonymous terms, 132 keywords with at least 15 occurrences were finally extracted. The incidence of the top 20 highest-frequency keywords occurrence was shown in Figure 8B, in which “pancreatic-cancer,” “gut microbiota,” “microbiome,” “colorectal-cancer,” and “inflammation” were the top five most commonly utilized keywords. The keywords overlay visualization was displayed in Figure 8C. In accordance to the color gradient in the bottom right corner, red dots like “pancreatic-cancer,” “gut microbiome,” “antibacterial,” “tumor microbiome,” “fusobacterium-nucleatum,” “dysbiosis” and “diversity” were recent keywords that seemed to represent the current research frontiers. In addition, we created keywords clustering analysis mappings via CiteSpace. There were 13 clusters as shown in Figure 8D, including #0breast cancer, #1pancreaticoduodenectomy, #2gut microbiota, #3pancreatic cancer, and #4periodontal disease, #5colon cancer, #6cells, #7efficacy, #8risk factors, #9pancreatic ductal adenocarcinoma, #10*escherichia coli*, #11*salmonella typhimurium* a1-r and #12green fluorescent. The values of modular, Q (0.7995) and mean silhouette, S (0.9401) were greater than 0.5. Any cluster was made up of many interconnected terms, and the smaller the number, the greater the number of keywords were included. In addition to quantitative analysis, this study also conducted qualitative analysis of keywords, Figures 9A,B showed the visualisation of high-frequency keywords for the periods of 2004 to 2013 and 2014 to 2023, respectively. By comparison, we summarised the new and increased frequency of keywords in the period from 2014 to 2023 as shown in Table 5. Figure 10A exhibits the top 25 keywords with the highest cited outbursts. The top three keywords among them with the highest outburst intensity were “*escherichia coli*,” “human pancreatic cancer,” and “*saccharomyces cerevisiae*” (14.71, 13.03, and 10.05, respectively). Interestingly, some bursting keywords such as “gemcitabine” (2020–2023), “tumor microenvironment” (2020–2023), “tumor microbiome” (2021–2023), “resistance” (2021–2023), “ductal adenocarcinoma” (2021–2023), “gut microbiome” (2021–2023), and “diagnosis” (2021–2023) are still in outbreak until 2023. In addition, this study also conducted a thematic evolutionary analysis using Sankey diagrams. Figure 10B showed the thematic progression of the three phases in the research of PC and GM.

**Table 5:** The qualitative analysis of keywords.

New Research Keywords in 2014–2023			Increased Keywords Popularity in 2014–2023		
Mechanism	Treatment	Diagnosis	Mechanism	Treatment	Diagnosis
Chain fatty-acids, bile-acids, butyrate, epithelial-mesenchymal transition, tumor microenvironment, carcinogenesis, *salmonella typhimurium* a1-r, t-cells, probiotics, pathway, oxidative stress, obesity, nf-kappa-b, metabolites, kras, macrophages, insulin-resistance, inflammatory-bowel-disease, high-fat diet, fusobacterium-nucleatum, diversity, dysbiosis, commensal bacteria, toll-like receptors, antibiotics	Resistance, chemoresistance, double-blind, bacterial therapy, autoimmune pancreatitis, ulcerative-colitis, targeted therapy, prognosis, meta-analysis, body-mass index	Potential role, biomarker, prognosis	Inflammation, angiogenesis, escherichia-coli, helicobacter-pylori infection, proliferation, metabolism, mechanisms, infection, gene-expression, epidemiology, risk-factors	Gemcitabine, gene-therapy, immunotherapy, metastasis, survival	Diagnosis

## 4 Discussion

The development and treatment of PC are intimately related to GM, according to an increasing number of studies (Sethi et al., 2018; Wei et al., 2019; Brandi et al., 2021; Liu et al., 2022). To date, the present study is the first bibliometric analysis on PC and GM. In this study, we analyzed 1,449 literatures from the WoSCC to summarize the trends and potential future research hotspots in the field during the past 20 years, hoping to provide an insightful perspective for future study.

The quantity of literatures is the most intuitive sign of recent developments in an area. In accordance with the findings in Figure 2, the quantity of literatures on PC/GM has commonly increased during the past 20 years. Roughly, it could be subdivided into three phases: 2004–2013 was the stable period, in which the annual number of literatures hardly fluctuated significantly, hovering between 30 and 40; 2014–2018 was the accelerated period, in which the quantity of articles increased significantly, and it was noteworthy that in 2018, more than 100 papers were published, which may be related to the continuous improvement of GM culture technology; 2019–2023 was the steep increase period, the number of literatures ascended quickly and peaked in 2022. The decrease in the number of articles in 2023 may be explained by the fact that the start of this study was in April 2023. By fitting the curve with a polynomial, we inferred that the total number of literatures in 2023 amounted to approximately 268. Of these, about 52.38% (*n* = 759) were released to the public within the last 5 years. The above findings may indicate that this area has drawn ongoing interest from academics and is a potential research hotspot. Therefore, we venture to guess that this area will keep continuing to grow quickly.

Regarding the country analysis, studies from 83 countries were enrolled in this study. Most of the high-producing countries were concentrated in North America, Asia, and Europe (Figure 3A). The results in Table 2 as well as Figure 3B showed that the USA, China, Japan, Germany, and Italy were the main countries studying PC and GM, with the total number of literatures greater than 100, among which, more over half of the total literatures came from the USA and China. According to statistics, the USA led the world in total literatures, H-index and total citations, far surpassing other countries. Obviously, the USA was the most prominent research force in the area. This may be due to the long and stable research base, abundant human resources and substantial financial support in the USA (Guo et al., 2023). Interestingly, China, as a developing country, had increased its publication rate almost exponentially in the last 5 years, especially in 2022 when the annual publication volume (*n* = 64) had overtaken the USA to become first place, while the total publication volume and H-index were far ahead of other developed countries to rank second, but the average number of cited articles was relatively low. This indicated that although China was late in participating in this research field, it had given increasing focus on it in the past few years, which was confirmed in the overlay visualization map in Figure 3D. This may be related to the large population base and strong research power in China as well as the increasing incidence of PC in China year by year (Liu et al., 2022). In terms of country collaboration, we can see from Figure 3C that points with the same color indicated a collaborative relationship, and the wider the connecting line between points, the higher the frequency of collaboration. That is to say, the USA, Japan, and China had the closest interaction, and France, the United Kingdom, India, and South Korea had some cooperation. In addition, Italy, Netherlands, Greece and Spain also had cooperative relations. A good cooperative relationship can promote the scientific research development of each country and advance the progress of this research field, thus producing a win–win situation. By analysing countries in this research area from different perspectives, it is possible for researchers to gain a more comprehensive understanding of which countries are currently in a major leading position and with whom relevant researchers can prioritise collaboration, such as the USA, Japan and China.

Regarding the institutional analysis, the top ten organizations were all from the United States, France and the United Kingdom (Table 3). Among them, institutions from the USA occupied 70%, which undoubtedly confirmed the strong research power of the USA and on the other hand, hinted at the imbalance of global academic resources. It was easy to see that the University of California System had made a great contribution in this field. Interestingly, although Harvard Medical School only ranked 10th in total papers, it ranked third in terms of average number of citations, which we believed may be due to the late start of research in this area. It was worthy of note that although there were no institutions from China in the top ten, the overlay visualization map of institutions in Figure 4B showed that institutions from China such as Sichuan University, Zhejiang University, Shanghai Jiao Tong University and Peking Union Medical College Chinese Acad Med Sci had larger AAY values, indicating that they had started to be active in the field in recent years. BC values in excess of 0.1 were found at six institutions (Figure 4A). There was little close cooperation and communication among academic institutions in various nations (Figure 4B), with the majority of institutional cooperation was only within countries. We suggest that institutions between countries should strengthen their cooperation, especially in developing countries. More than half of the top ten funded institutions came from the United States and Japan (Figure 4C), and financial support is also crucial in scientific research. In conclusion, by conducting the institutional analysis from different perspectives, the patterns of close collaboration between the many institutions were revealed, which facilitated the researcher to find more suitable collaborating institutions in the field.

Regarding the author co-occurrence analysis, as can be seen in Figures 5A,B, the author with the most articles and the highest H-index was Prof. Robert M. Hoffman from AntiCancer Inc., followed by Zhao Minglei and Zhang Yong. Prof. Robert M. Hoffman focused on the effect of *Salmonella typhimurium* A1-R on PC (Nagakura et al., 2009; Hiroshima et al., 2013; Kawaguchi et al., 2018; Murakami et al., 2018, 2019). Since 2009, he successively found that *Salmonella typhimurium* A1-R could inhibit the metastasis of PC (Hayashi et al., 2009; Nagakura et al., 2009; Yam et al., 2010). After establishing animal models of experimental lymph node metastasis, experimental lung metastasis, and spontaneous lymph node metastasis from PC, they observed eradication of metastases with no adverse effects by treatment with *Salmonella typhimurium* A1-R injection for 7–21 days. This led to the discovery of the clinical potential of curing cancer metastases by targeting *Salmonella typhimurium* A1-R alone without toxic chemotherapy (Hayashi et al., 2009; Nagakura et al., 2009). Immediately after, in 2010, they found that *Salmonella typhimurium* A1-R inhibited liver metastasis from PC (Yam et al., 2010). Since then, they had focused their research on the treatment of PC. In an experimental study published in J Cell Biochem, *Salmonella typhimurium* A1-R was identified to inhibit tumor development and metastasis in a mouse model of PC through stimulating tumor infiltration of tumor-killing CD8 T cells (Murakami et al., 2018). *Salmonella typhimurium* A1-R in conjunction with gemcitabine (GEM) had also shown good results in treating patients with GEM-resistant pancreatic cancer (Kawaguchi et al., 2018). Notably, Dr. George Miller was the only one with more than 100 citations on average, although he ranked only fifth in terms of number of literatures. This may be since six of George Miller’s relevant papers were published in Q1 journals (Fan et al., 2018; Pushalkar et al., 2018; Aykut et al., 2019; Leinwand and Miller, 2019; Sethi et al., 2019; Vitiello et al., 2019). Among them, a study they published in *Nature* in 2019 found that fungal community infiltrating pancreatic ductal adenocarcinoma (PDA) tumors was significantly enriched for Malassezia spp. and that they could promote PDA by activating mannose-binding lectin (MBL)-driven complement cascades (Aykut et al., 2019). An article from *Nat Rev Clin Oncol* suggested that the microbiota could be considered a predictive biomarker for risk stratification in PDA patients (Leinwand and Miller, 2019). Regarding author co-citation analysis, Michaud DS had the most co-citations with 361, ahead of Pushalkar as well as Riquelme. It showed that their research on PC and GM had received wide attention and generated high impact, therefore, an author’s academic impact cannot be determined only by the quantity of papers they have made, as many factors influence an article’s frequency of being cited. In conclusion, each of these scholars had an important contribution to make to the field of PC and GM, and they are likely to continue their achievements in the field.

Journal analysis is an important part of bibliometric research, which can help researchers quickly select the most appropriate target journals (Liu et al., 2022). Table 4 summarized key information about the top 10 journals, most of which were general journals, divided into four main categories: oncology, immunology, gastrointestinal journals, and nutrition. *Cancers*, *International Journal of Molecular Science* and *Onco Targets* and *Therapy journals* were the three most popular journals. Therefore, it would be more probable for studies on PC and GM to get published in these literatures. It was noteworthy that none of the journals were assigned a Q1 classification, which implied that the field still needs a lot of effort to be drilled. Regarding the analysis of journal co-citations, *Nature*, *Science* and *Cancer Res Journals* were the three most co-cited journals (Figure 6B), and we can boldly speculate that these journals will be more inclined to publish high-quality research outcomes. In addition, the two citation paths in Figure 6C showed that studies published in *Molecular/Biology/Genetics* or *Health/Nurse/Medicine* Journals were usually cited in *Lar/Biology/Immunology* and *Cine/Medical/Clinical* Journals. In summary, the current research on PC and GM is mainly focused on basic research and translational medicine.

Citation analysis allows evaluation of the actual scholastic influence of literature in a given area and reflects current research priorities (Liu et al., 2022). Supplementary Table S1 summarized the top ten most cited papers, which discussed the application of GM for PC screening and therapy. Eight of them were articles and the remaining two were reviews, which allowed young scholars to get a quick grasp of this research area. The paper published in Science by Leore et al. (Aykut et al., 2019) had the most citations (Geller et al., 2017), which observed that bacteria are a part of the PDA tumor microenvironment as well as that all species expressing the long isoform of the bacterial enzyme cytidine deaminase (CDD_L_) conferred gemcitabine resistance, while 98.4% of CDD_L_-containing species pertained to Gammaproteobacteria class. References that have been heavily cited by other research over a while are called strong citation bursts, which can reflect trends in the popularity of a particular field (Cheng et al., 2022a). As shown in Figure 7C, the first burst originated with an article published by Farrell et al. in 2012 (Geller et al., 2017), which first observed an association between salivary microbiota and PC, generating inspiration for the study of GM and PC, as they were related to some extent (Kageyama et al., 2023). In addition, Mitsuhashi et al. published the study with the highest burst intensity in 2015. They found that while there was no connection between Clostridium spp. status and molecular alterations in PC, Clostridium tumefaciens status was independently associated with poorer prognosis in PC and Clostridium tumefaciens status might be employed as an indicator of prognosis in PC (Mitsuhashi et al., 2015).

Through reference timeline analysis, keyword clustering analysis, keywords burst analysis and keywords qualitative analysis, the current research frontiers and hotspots are roughly in the following three areas: (1) mechanistic studies on the involvement of GM in PC; (2) the utilitarian function of GM in the diagnosis of PC; (3) the impact of GM on the treatment of PC.

An increasing quantity of studies had identified that GM was involved in the development of PC. Firstly, GM could influence the development of PC through inflammatory response, and “inflammation” had received increasing attention in the last decade compared to the previous decade. Chronic pancreatitis was associated with a much higher incidence of PC than healthy people (Hsieh et al., 2023). Some studies had found that metabolites of GM could motivate inflammatory reactions, such as the lipopolysaccharide (LPS) (Daniluk et al., 2012). Chronic inflammation could activate KRAS mutations in pancreatic endocrine cells and induce functional epithelial cell dedifferentiation, thus leading to PDA (Gidekel Friedlander et al., 2009). Short-chain fatty acids (SCFA) were fermentation products of GM which include butyrate, acetate, and propionate. Several studies found that butyrate had an anti-inflammatory effect on PC and also inhibited cancer cell growth and promoted apoptosis (Natoni et al., 2005; Attebury and Daley, 2023). Knorr et al. found that *H. pylori* could secrete cytotoxin-associated proteins, which promoted chronic inflammatory oxidative stress, triggering cellular carcinogenesis (Knorr et al., 2019). However, the relationship between *H. pylori* infection and PC remains controversial. Wei et al. found a high incidence of *H. pylori* infection and positivity of *H. pylori* serum antibodies in PC (Wei et al., 2019), whereas Kumar et al. observed a very low prevalence of *H. pylori* in PC (Kumar et al., 2020). There is a need for more studies to explain this contradiction in the future. Secondly, GM was involved in the immune response to influence PC development, including innate immunity and acquired immunity. A study published in 2018 by Miller George et al. found that bacterial ablation was linked to immunogenic reprogramming of the PDA tumor microenvironment, involving reduced myeloid-derived suppressor cells as well as increased differentiation of M1 macrophages, enhancing CD1+ T cell TH4 differentiation and the activation of CD8+ T cells, demonstrating that the apparent dysbiosis of bacterial ecology associated with PDA leads to innate and adaptive immune suppression (Pushalkar et al., 2020). In addition, another study found that GM stimulates PDA tumor development by regulating the intratumoral infiltration and activation of NK cells through the construction of an immunodeficiency model and an immune function model (Yu et al., 2022). Thirdly, GM engaged in the catabolism of bile acids which influenced the development of PC. Several existing studies had found that bile acids could promote PC invasion by disrupting the bilayer of lipid molecules in contact with cells (Shrader et al., 2021). Zampa et al. found that GM could hinder tumorigenesis by inhibiting the conversion of primary bile acids towards secondary bile acids (Zampa et al., 2004). Although there was evidence of the involvement of GM in the development of PC, the exact mechanisms had not been elucidated and more focused research was needed in the future.

Microbiome analysis may have the capacity to be a quick, non-invasive biomarker for pancreatic cancer diagnosis, prognosis, and prediction. A prospective study from China found that A prospective study from China found that patients with PDA had significantly lower gut microbial diversity with significantly higher levels of Bacteroidetes and lower levels of Firmicutes and Proteobacteria compared to healthy controls (Ren et al., 2017). Notably, some studies had found no significant difference in alpha diversity between PC and healthy people (Half et al., 2019; Zhou et al., 2021), so more research was required to explore this problem for the future. As reported by Pushalkar et al., PDA patients had considerably greater relative abundances of Aspergillus, Ascomycetes, and Eubacterium in their stools than those healthy controls (Pushalkar et al., 2018). One study found little overlap between fecal microbiome features rich in PDA and those rich in other cancer types (e.g., colorectal cancer) by building multiple predictive models, and when these microbiomes were combined with CA199, the diagnostic accuracy was improved (Kartal et al., 2022). This provided ideas for non-invasive diagnosis of PC using microbial markers. In addition to cancer diagnosis, the microbiome can also be used as a prognostic marker for PC. For instance, a prior study in a US cohort revealed greater alpha diversity in the tumor microbiome of long-term survivors compared to short-term survivors (Riquelme et al., 2019). Yang et al. identified Streptococcus as a potential marker for predicting PC (AUC = 0.927) and PC liver metastasis (AUC = 0.796) by constructing a random forest model as well as plotting ROC curves (Yang et al., 2023). These studies showed the promise of GC as an early, noninvasive, and accurate detection tool for PC, thereby reducing patient suffering and healthcare costs. However, many relevant studies had been conducted at the microbiological level, so more animal experimentation and clinical trials are needed to explore and validate the diagnostic potential of PC in the future.

There are very limited treatment options for pancreatic cancer, with gemcitabine (GEM) being the main chemotherapeutic agent. Studies on the involvement of GM in the efficacy of PC treatment had been conducted (Sethi et al., 2019; Zhang and Tang, 2022; Seo and Wargo, 2023). It had been shown that GM can influence the efficacy of chemotherapy for PC. Toshikatsu Okumura et al. created a xenograft tumor model that was treated with *Lactobacillus casei* and discovered that it boosted the effectiveness of 5-FU and cisplatin by inducing p53-mediated activation of apoptosis (Kita et al., 2020). Sandra Liekens et al. found that pyrimidine nucleoside phosphorylase, an enzyme produced by mycoplasma, mediated the phosphorylation of uridine, 2′-deoxyuridine, and thymidine and reduced nucleoside chemotherapy anticancer outcomes (Vande Voorde et al., 2014). In addition, the microbiota’s metabolites can influence the efficiency with which a pancreatic cancer therapy. Several studies had found that Indole-3-Acetic Acid (3-IAA), a tryptophan metabolite generated by the GM, enhanced the efficacy of chemotherapy against PC (Trotter et al., 1987; Seo and Wargo, 2023). In addition, the research hotspot that GM could influence chemotherapy resistance had begun to attract widespread attention in the last decade. For example, the treatment of patients with GEM-resistant PC was enhanced when *Salmonella typhimurium* A1-R was added (Kawaguchi et al., 2018). Although immunotherapy had shown remarkable success in malignant tumor, it had so far been ineffective in PC. Hence, there is an urgent need to develop effective methods of treatment. Some studies had demonstrated the potential for GM to alter immunotherapy in PC in besides influencing the effectiveness of chemotherapy (Riquelme et al., 2018; Zarour, 2022). According to research by Rahul et al., the gut microbial-derived metabolite trimethylamine N-oxide (TMAO) enhanced the efficacy of PC by directly driving in macrophages the immunostimulatory phenotype and enhanced the response of PC to immune checkpoint blockade (Mirji et al., 2022). “Bacterial therapy” also came to the researchers’ attention, and fecal microbial transplantation (FMT) was one of the most common types of it. FMT had been used in the treatment of ulcerative colitis and *Clostridium difficile* infections, but had not yet matured in the treatment of PC. A study by Riquelme et al. found that FMT was effective in influencing tumor growth and immune infiltration in a mouse model of PC (Riquelme et al., 2019). GM combined with immunotherapy may be an opportunity for effective treatment, but there were fewer studies on this. We hope that more clinical trials can be conducted with reference to existing promising animal experiments in the near future, which may revolutionize the treatment of PC.

## 5 Strength and limitations

This study has the following advantages: it is the first comprehensive bibliometric analysis of the pancreatic cancer and gut microbiota research domain. The data were visualized as comprehensively as possible with the help of eight tools (Microsoft Excel 2021, CiteSpace, VOSviewer, Scimago Graphica, Graphpad Prism, Origin, the R package “bibliometrics” and the bibliometric online analysis platform). In addition, keywords were censored and merged to reduce meaningless interference in this study. However, this study still has some limitations: Firstly, the WoSCC database alone was searched for studies, so some studies published only in other databases (Scopus, PubMed, Embase, etc.) may have been missed. It is worth noting that WoSCC is generally considered to have high-quality and complete data and is a common tool for bibliometric analysis (Cheng et al., 2022b; Jiang et al., 2023), so we do not think this will have a significant impact on the overall trend. Secondly, some non-English studies may be missed due to language limitations. Finally, although we tried to make the search strategy as complete and exhaustive as possible, there may be some selection bias due to the diversity of keywords. Despite these limitations, this study still reflects the research tendencies and hot spots in the area of PC and GM.

## 6 Conclusion

In a word, this research has performed a bibliometric visualization of the field of PC and GM using tools such as CiteSpace, VOSviewer, as well as a bibliometric online analysis platform. There has been a rise in interest in this field during the past 20 years. So far, the USA, Japan and China are undoubtedly leading in this area while there is a demand to improve collaboration among international institutions. The mechanisms of GM involved in pancreatic carcinogenesis, the potential of GM as a marker for diagnosis and prognosis of PC, and the influence of GM on the treatment of PC have been the hotpots and frontiers of research in recent years. This study summarizes the present status of global study on PC and GM and also provides effective suggestions and ideas for future research.

## Author contributions

ShW: Conceptualization, Data curation, Methodology, Software, Visualization, Writing – original draft. SuW: Conceptualization, Writing – review & editing. KA: Writing – review & editing. LX: Data curation, Writing – review & editing. HZ: Writing – review & editing. YN: Conceptualization, Writing – review & editing. TY: Writing – review & editing.

## Footnotes

^1^https://bibliometric.com
