# Supplementary material for: Bibliometric analysis of global research trends between gut microbiota and pancreatic cancer: from 2004 to 2023
Source: Front Microbiol. 2023 Nov 27;14:1281451. doi: 10.3389/fmicb.2023.1281451 (PMC10715435; doi:10.3389/fmicb.2023.1281451)
Supplement: Supplementary file 1 [file Table_1.DOCX]

**Supplementary Table1** Top ten highly cited studies.

| **Rank** | **Title** | **First author** | **Year** | **Type** | **TC** | **Journal** | **IF (2022)** |
| --- | --- | --- | --- | --- | --- | --- | --- |
| 1st | Mucins in the mucosal barrier to infection | Linden, S. K | 2008 | Review | 763 | Mucosal Immunology | 8.701 |
| 2nd | Potential role of intratumor bacteria in mediating tumor resistance to the chemotherapeutic drug gemcitabine | Geller, Leore T | 2017 | Article | 725 | Science | 63.714 |
| 3rd | Capillary electrophoresis mass spectrometry-based saliva metabolomics identified oral, breast and pancreatic cancer-specific profiles | Sugimoto, Masahiro | 2010 | Article | 657 | Metabolomics | 4.474 |
| 4th | The human tumor microbiome is composed of tumor type-specific intracellular bacteria | Nejman, Deborah | 2020 | Article | 627 | Science | 63.714 |
| 5th | The Pancreatic Cancer Microbiome Promotes Oncogenesis by Induction of Innate and Adaptive Immune Suppression | Pushalkar, Smruti | 2018 | Article | 573 | Cancer Discovery | 38.272 |
| 6th | Tumor Microbiome Diversity and Composition Influence Pancreatic Cancer Outcomes | Riquelme, Erick | 2019 | Article | 555 | Cell | 66.850 |
| 7th | Obesity and cancer risk: Emerging biological mechanisms and perspectives | Avgerinos,Konstantinos, I | 2019 | Review | 473 | Metabolism-Clinical and Experimental | 13.934 |
| 8th | In Vitro Expansion of Human Gastric Epithelial Stem Cells and Their Responses to Bacterial Infection | Bartfeld, Sina | 2015 | Article | 456 | Gastroenterology | 33.883 |
| 9th | Variations of oral microbiota are associated with pancreatic diseases including pancreatic cancer | Farrell, James J | 2012 | Article | 388 | Gut | 31.793 |
| 10th | Human oral microbiome and prospective risk for pancreatic cancer: a population-based nested case-control study | Fan, Xiaozhou | 2018 | Article | 383 | Gut | 31.793 |
